# Registering Diplomas in Pennsylvania

**Published:** 1894-01

**Authors:** 


### REGISTERING DIPLOMAS IN PENNSYLVANIA.

Many complaints have been received in regard to the inequality of charges made by the recorders of different counties for registration, these ranging from one to three dollars.

As far as known, the law does not regulate this, and it is presumed no power exists to make the fees equal throughout the State. It would seem to be the duty of the State Board to remedy this injustice by proposing an amendment to the law, if that be the best way of reaching the difficulty.

